# Cancer care in German centers of excellence during the first 2 years of the COVID-19 pandemic

**DOI:** 10.1007/s00432-022-04407-1

**Published:** 2022-10-14

**Authors:** Volker Arndt, Daniela Doege, Stefan Fröhling, Peter Albers, Hana Algül, Ralf Bargou, Carsten Bokemeyer, Martin Bornhäuser, Christian H. Brandts, Peter Brossart, Sara Yvonne Brucker, Tim H. Brümmendorf, Hartmut Döhner, Norbert Gattermann, Michael Hallek, Volker Heinemann, Ulrich Keilholz, Thomas Kindler, Cornelia von Levetzow, Florian Lordick, Ulf Peter Neumann, Christoph Peters, Dirk Schadendorf, Stephan Stilgenbauer, Thomas Zander, Daniel Zips, Delia Braun, Thomas Seufferlein, Gerd Nettekoven, Michael Baumann

**Affiliations:** 1 Division of Clinical Epidemiology and Aging Research (C070), Unit of Cancer Survivorship (C071), German Cancer Research Center (DKFZ), Im Neuenheimer Feld 280, 69120 Heidelberg, Germany; 2 Division of Translational Medical Oncology, German Cancer Research Center (DKFZ), National Center for Tumor Diseases (NCT), Heidelberg, Germany; 3 Department of Urology, Comprehensive Cancer Center/Center for Integrated Oncology (CIO Aachen, Bonn, Cologne, Düsseldorf), Medical Faculty of the Heinrich Heine University Düsseldorf, Düsseldorf, Germany; 4 Comprehensive Cancer Center TUM (CCCMTUM), Klinikum Rechts der Isar of the Technical University of Munich, Munich, Germany; 5 Comprehensive Cancer Center Mainfranken, University Hospital Würzburg, Würzburg, Germany; 6 Medical Clinic and Polyclinic II, Center for Oncology, University Cancer Center Hamburg, University Medical Center Hamburg-Eppendorf, Hamburg, Germany; 7 Medical Clinic I, National Center for Tumor Diseases (NCT/UCC), University Hospital Carl Gustav Carus Dresden, TU Dresden, Dresden, Germany; 8 University Cancer Center (UCT) Frankfurt-Marburg, Frankfurt University Hospital, Frankfurt, Germany; 9 Medical Clinic III and Center for Integrated Oncology (CIO Aachen, Bonn, Cologne, Düsseldorf), University Hospital Bonn, Bonn, Germany; 10 Comprehensive Cancer Center Tübingen-Stuttgart and Department of Women’s Health, University Hospital Tübingen, Tübingen, Germany; 11 Medical Clinic IV and Center for Integrated Oncology (CIO Aachen, Bonn, Cologne, Düsseldorf), University Hospital of RWTH Aachen, Aachen, Germany; 12 Comprehensive Cancer Center Ulm (CCCU) and Department of Internal Medicine III, University Hospital Ulm, Ulm, Germany; 13 Department of Hematology, Oncology and Clinical Immunology, Comprehensive Cancer Center/Center for Integrated Oncology (CIO Aachen, Bonn, Cologne, Düsseldorf), Medical Faculty of the Heinrich Heine University Düsseldorf, Düsseldorf, Germany; 14 Clinic I for Internal Medicine and Center for Integrated Oncology (CIO Aachen, Bonn, Cologne, Düsseldorf), University Hospital Cologne, Cologne, Germany; 15 Medical Clinic and Polyclinic III, LMU Hospital, Munich, Germany; 16 Charité Comprehensive Cancer Center (CCCC), Berlin, Germany; 17 University Cancer Center (UCT), University Medical Center Mainz, Mainz, Germany; 18 University Cancer Center Leipzig and Department of Medicine II, University of Leipzig Medical Center, Leipzig, Germany; 19 Department of Visceral and Transplantation Surgery, University Hospital of RWTH Aachen, Aachen, Germany; 20 Tumor Center Freiburg, Institute of Molecular Medicine and Cell Research, Freiburg, Germany; 21 West German Tumor Center (WTZ) Essen and Clinic for Dermatology, Essen University Hospital, Essen, Germany; 22 Comprehensive Cancer Center Tübingen-Stuttgart and Department of Radiation Oncology, University Hospital Tübingen, Tübingen, Germany; 23 German Cancer Research Center (DKFZ), Heidelberg, Germany; 24 German Cancer Society, Berlin, Germany; 25 Department of Internal Medicine I, University Hospital Ulm, Ulm, Germany; 26 German Cancer Aid, Bonn, Germany

**Keywords:** SARS-CoV-2 infection, Early diagnosis of cancer, Treatment delays, Aftercare, Comprehensive cancer centers

## Abstract

**Purpose:**

An increasing number of international studies demonstrate serious negative effects of the COVID-19 pandemic on the timely diagnosis of cancer and on cancer treatment. Our study aimed to quantitatively and qualitatively evaluate the capacities of German Comprehensive Cancer Centers (CCCs) in different areas of complex oncology care during the first 2 years of the COVID-19 pandemic.

**Methods:**

Prospective panel survey over 23 rounds among 18 CCCs in Germany between March 2020 and June 2022.

**Results:**

The COVID-19 pandemic substantially affected the oncological care system in Germany during the first 2 years. Persistent limitations of care in CCCs primarily affected follow-up (− 21%) and psycho-oncologic care (− 12%), but also tumor surgery (− 9%). Substantial limitations were also reported for all other areas of multidisciplinary oncological care.

**Conclusions:**

This study documents the limitations of oncological care during the COVID-19 pandemic and highlights the need to develop strategies to avoid similar limitations in the future.

## Background

Globally, health services for noncommunicable diseases have been severely compromised since the onset of the COVID-19 pandemic (World Health Organization 2020). An increasing number of studies from various European countries (Aapro et al. 2021; Dinmohamed et al. 2020a, b; Kleemann et al. 2022; Maluchnik et al. 2021; Maringe et al. 2020; Skovlund et al. 2021; Weisel et al. 2020), the USA (Aapro et al. 2021; Kaufman et al. 2020, 2021), and other countries (Aapro et al. 2021; Marques et al. 2021; Mathelin et al. 2021) report that the COVID-19 pandemic had a serious negative impact on the timely diagnosis of cancer and on cancer treatment, including a significant decrease in new cancer diagnoses. With the classification of the COVID-19 spread as a pandemic by the WHO on 11 March 2020, far-reaching measures were imposed worldwide to contain the spread of SARS-CoV-2 and to prevent an overload of health systems with critically ill COVID-19 patients. In many countries, health authorities advised hospitals and health facilities to defer medical care for non-acute or non-life-threatening conditions and to postpone cancer screenings while the pandemic was being addressed (Erdmann et al. 2021).

The aim of the present evaluation is to provide a quantitative and qualitative inventory of the capacities of German centers of excellence in oncology in various areas of complex oncological care in the period from March 2020 to June 2022.

## Methods

**Fig. 1:**
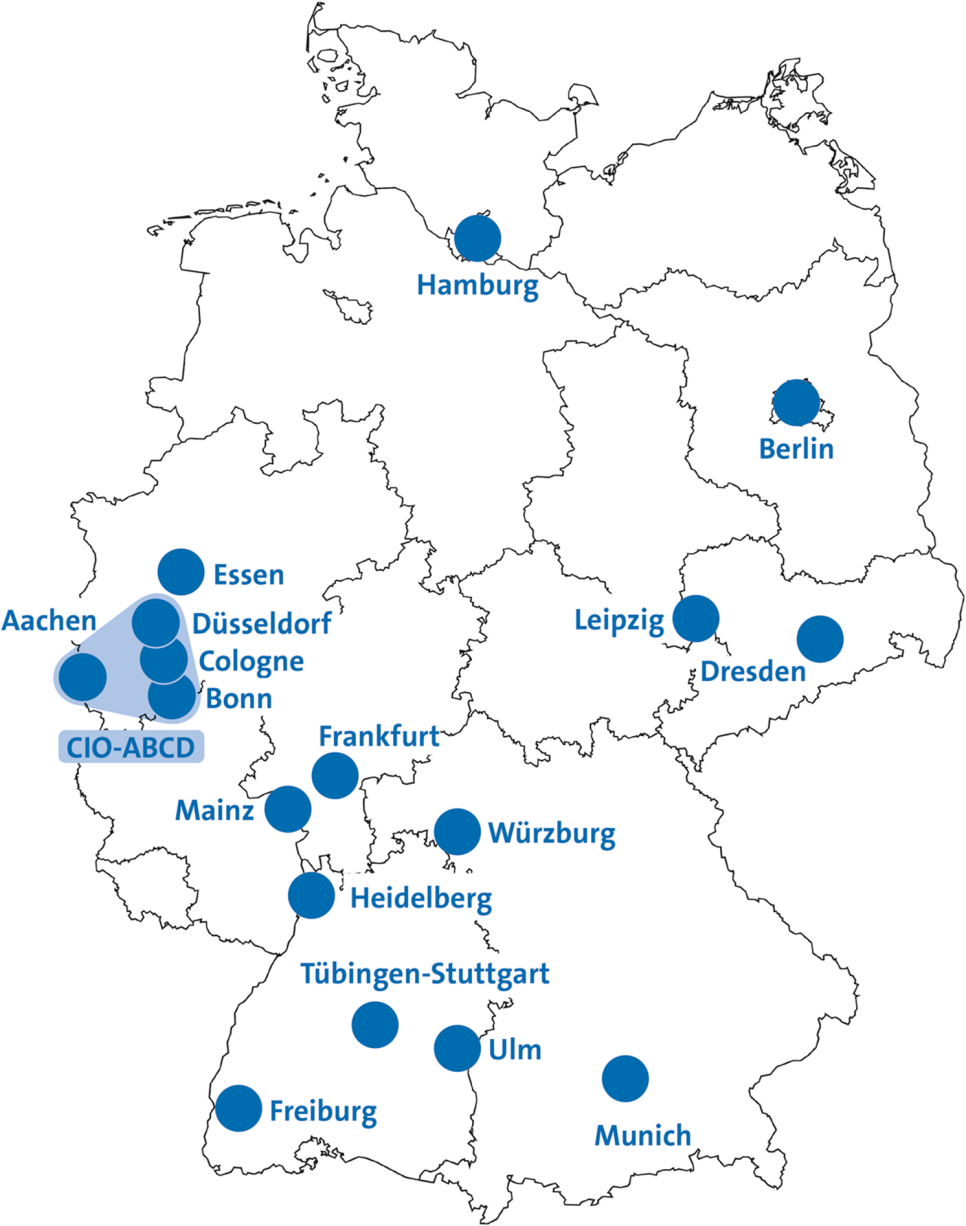
Comprehensive Cancer Centers’ locations participating in the study (*Clinics in North Rhine-Westphalia were not included due to strike-related staff absences in May/June 2022)

Concerned about the possible impact of the COVID-19 pandemic on the utilization and supply of oncological care, a task force was set-up by the German Cancer Research Center (DKFZ), German Cancer Aid, and the German Cancer Society (DKG) in March 2020. The task force established a prospective panel study among 18 Comprehensive Cancer Centers (CCCs) to early detect deficits in cancer care capacities driven by the COVID-19 pandemic and to inform decision-makers and the public. The 18 participating CCCs (Fig. 1) care for almost 20% of the 500,000 incident cancer cases in Germany per year (Klein 2018).

Between March 2020 and June 2022, the directors of the participating CCCs (or their representatives) were regularly asked via a standardized questionnaire about the extent of potential limitations (and the certainty of each rating) in various domains of oncology care:

- Diagnostics (imaging)
- Diagnostics (pathology and laboratory)
- Tumor biopsies
- Tumor board
- System therapy of solid tumors
- System therapy of hematological tumors
- Radiotherapy
- Tumor surgery
- Pediatric oncology
- Psychooncology/nutrition and exercise therapies/social counseling service
- Special offers (e.g., fertility protection)
- Aftercare
- Palliative care
- Information hotline

The questionnaire was initially administered as a questionnaire-based telephone interview, subsequently as an e-mail (April–August 2020) and online survey (November 2020–June 2022). Up to three reminders as well as prompt feedback on results were sent to the clinic representatives in each round of the survey to achieve high response. Overall, over 23 rounds were applied.

The questionnaires were checked for plausibility after return. Any implausible data were resolved after consultation with the respective clinic representatives. Per round, capacity of cancer care per domain was calculated as unweighted average of all reported care capacities for the particular domain. The centers’ capacities before the onset of the COVID-10 pandemic were considered as reference for evaluation (100% capacity).

The responses from the CCCs in North Rhine-Westphalia for May and June 2022 were excluded from the analysis due additional capacity restrictions caused by a strike of health care workers in university hospitals in North Rhine-Westphalia.

## Results

The COVID-19 pandemic has led to long-lasting and significant limitations in oncologic care, primarily in the areas of aftercare, psycho-oncology and tumor surgery (Table 1). Over the entire study period and across all participating CCCs, the provision of care was reduced by 21% in the area of aftercare, by 12% in psycho-oncological care, and by 9% with respect to tumor surgery compared to the time "before Corona". At the beginning of the first wave (March/April 2020), restrictions of about 70% in follow-up care, 32% in psycho-oncology and 20% in tumor operations were reported. Substantial restrictions were also observed in all other examined sectors of oncological care, especially in the care of hematological neoplasms with intensive therapies (i.e., acute leukemia and lymphoma with high-dose chemotherapy and autologous peripheral blood stem cell transplantation, as well as allogeneic stem cell transplantation).

**Table 1:**
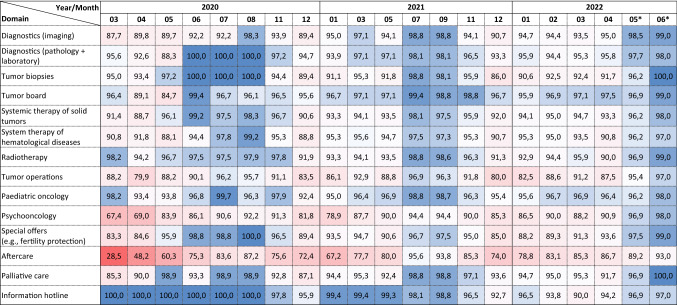
Capacity of oncological care by domain and time point (compared to capacity before March 2020)

Over the 28-month observation period, there were seasonal fluctuations in care capacity with higher capacities during the summer months and peaks of restrictions in the winter and spring months (Table 2). Across different epidemiological parameters describing the COVID-19 situation in Germany (i.e., number of reported new COVID-19 cases (Robert Koch-Institute 2022b), COVID-19 hospitalization rate [Robert Koch-Institute 2022a), utilization of intensive care units by COVID-19 cases (Robert Koch-Institute, Divi E.V. 2022)] the utilization of intensive care beds was most strongly associated with restrictions in oncological care across different domains (Table 3).

**Table 2:**
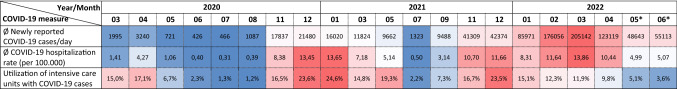
Number of reported new COVID-19 cases (Robert Koch-Institute 2022b), COVID-19 hospitalization rate (Robert Koch-Institute 2022a) and utilization of intensive care units in Germany (Robert Koch-Institute, Divi E.V. 2022) during the study period

**Table 3:** Association between number of reported new COVID-19 cases (Robert Koch-Institute 2022b), COVID-19 hospitalization rate (Robert Koch-Institute 2022a), utilization of intensive care units in Germany (Robert Koch-Institute, Divi E.V. 2022) and domain specific capacity of oncological care

Domain of oncologic care	Ø newly reported COVID-19 cases/day	Ø hospitalization rate (per 100.000)	Utilization of intensive care units with COVID-19 cases
Diagnostics (imaging)	0.09	− 0.10	− 0.45
Diagnostics (pathology + laboratory)	− 0.16	− 0.35	− 0.51
Tumor biopsies	− 0.37	− 0.78	− 0.87
Tumor board	0.17	0.15	− 0.11
System therapy of solid tumors	− 0.09	− 0.47	− 0.78
System therapy of hematological diseases	− 0.08	− 0.30	− 0.49
Radiotherapy	− 0.32	− 0.67	− 0.67
Tumor operations	− 0.11	− 0.47	− 0.75
Pediatric oncology	0.00	− 0.41	− 0.53
Psychooncology	0.21	− 0.05	− 0.52
Special offers (e.g., fertility protection)	− 0.22	− 0.39	− 0.71
Aftercare	0.29	0.09	− 0.42
Palliative care	0.01	− 0.29	− 0.63
Information hotline	− 0.86	− 0.73	− 0.25

With the exception of follow-up care (− 25%), modest deficits (5–10%) were reported for most sectors of oncologic care at the beginning of 2022, despite very high numbers of new SARS-CoV-2 infections. In spring 2022, medical care was severely hampered by frequent absence of medical and nursing staff due to quarantine and self-isolation. Here, up to 10–20% absence of the regular staff and limited capacities for admitting further patients were reported.

At the beginning of spring 2022, there was a recovery in care capacity and most clinics were able to regain almost the pre-pandemic capacity in June 2022, despite the persistence of substantial staffing and organizational restrictions and corresponding additional compensatory expenditure. However, concerns have also been expressed that the lack of personnel will drain resources and could lead to renewed restrictions if the number of infections rises again.

## Discussion

The COVID-19 pandemic has led to measurable changes in oncological care in Germany (Rückher et al. 2022; Weisel et al. 2020) as well as in many other countries (Richards et al. 2020). A task force led by German Cancer Research Center, German Cancer Aid and German Cancer Society addressed this problem at an early stage and initiated the study presented here, which is certainly a remarkable effort also in international comparison. Considerable limitations were found in the area of aftercare and in psycho-oncological care, but also in tumor operations. These limitations affected almost all CCCs and they persisted over a long time of the observation period.

In retrospect, three different phases with restrictions in oncological care during the Corona pandemic can be described so far: The first phase was characterized by patients' concerns about infecting themselves when visiting the doctor, by contact restrictions to reduce the infection rate and to relieve the medical care system, and by hygiene-related organizational measures in outpatient and inpatient care. The second phase followed with an impending bottleneck in care capacities in intensive care units (from winter 2020), which led to a "backlog" in oncological care. The third phase mainly involved restrictions triggered by an acute, inter-professional staff shortage due to quarantine and self-isolation in view of very high rates of new infections from December 2021.

The medical care system has reacted to the new challenges. In addition to hygiene-related organizational measures, which inevitably tend to lead to a reduced patient throughput, more interdisciplinary case discussions (tumor conferences) were conducted via video. In follow-up care and in the area of psycho-oncology/nutritional and exercise therapies/social counseling, parts of the care capacity could be maintained via offering counseling on the telephone or in video conferences. In the case of systemic tumor therapies, therapy cycles were modified if clinically justifiable. Changes in hematological systemic therapies mainly involved a reduction or postponement of autologous and allogeneic transplants or cellular therapies.

The extent of the restrictions on tumor operations that we have described corresponds very well with previously published billing figures for Germany (Günster et al. 2020). The evaluation of the billing data also shows differences depending on the type of tumor. For example, whereas the number of colorectal cancer operations declined by 20 percent from 2019 to 2020 (presumably due to a decline in colorectal cancer screening and diagnosis), the number of breast cancer operations increased by 5% in the same period despite a temporary shutdown of the mammography screening program in 2020. This discrepant finding might be related to changes in breast cancer management during the COVID-19 pandemic. Primary systemic therapy (i.e., chemotherapy) was used less frequently due to increased susceptibility to Covid-19 complications, while at the same time the proportion of patients undergoing primary surgery increased (Gasparri et al. 2020).

The study presented here does not claim a representative, cross-sectoral result for the entire oncological care in Germany. But it demonstrates impressively the long-term, undesirable "side effects" of prioritizing available medical treatment capacities to only one sector. In addition to this prioritization, restrictions in capacity during the pandemic were also caused by further problems, e.g. long quarantine periods for clinical staff after virus exposure, less space in outpatient clinics or on wards due to spacing rules and other logistic challenges. Even though the figures presented here are based on self-reports by the participating CCCs, a high degree of validity can be assumed. In 72% of the statements, the degree of certainty of the correctness of the respective answer was self-rated as "very certain" and in 26% as "relatively certain".

The extent to which the pandemic-related restrictions described in this paper will have a long-term negative impact on treatment, on stage distribution in cancer diagnosis (with a shift towards more advanced cancer stage), and on survival cannot yet be quantified. On the part of the care providers, great efforts have been made to maintain care at a high level. More worrying, however, is a slump in the number of new cancer diagnoses due to suspended screening and delayed diagnostic clarification. First evaluations of regional cancer registries show a decline especially in the early stages of cancer (Voigtlander et al. 2021).

In conclusion, the oncological care system in Germany was substantially impaired during the first 2 years of the COVID-19 pandemic. Restrictions in oncological care provided by the CCCs were primarily related to follow-up care, psycho-oncology, but also tumor surgery and intensive system therapeutic therapies for hematological neoplasms. In addition to the observed limitations in oncological care, the effects of delayed diagnostic work-up have to be considered and strategies need to be developed to avoid such delays in the future.

## Data Availability

The datasets generated during and/or analyzed during the current study are available from the corresponding author on reasonable request.
